# Cancer Survivors’ Evolving Perceptions of a New Supportive Virtual Program

**DOI:** 10.3390/curroncol29110664

**Published:** 2022-11-05

**Authors:** Alexandra Robb, Tyler L. Brown, Andrew Durand, Carmen G. Loiselle

**Affiliations:** 1 Ingram School of Nursing, Faculty of Medicine and Health Sciences, McGill University, Montreal, QC H3A 2M7, Canada; 2 Department of Oncology, Faculty of Medicine and Health Sciences, McGill University, Montreal, QC H4A 3T2, Canada; 3 Department of Psychology, Faculty of Arts and Sciences, Concordia University, Montreal, QC H4B 1R6, Canada; 4 Lady Davis Institute for Medical Research, Jewish General Hospital, Montreal, QC H3T 1E2, Canada; 5 Segal Cancer Centre, Jewish General Hospital, Montreal, QC H3T 1E2, Canada

**Keywords:** cancer survivors, cancer care, digital health, social networks, survivorship programs, internet-based interventions, supportive care

## Abstract

This qualitative study begins to explore cancer survivors’ evolving perceptions of “Focus on the Future,” a 6-week supportive virtual program led by trained volunteers and health care professionals. Through purposive sampling, participants (n = 10) enrolled in the program were individually interviewed shortly before attending, mid-way through, and at program completion. Interviews were digitally recorded and transcribed verbatim. Thematic analysis was used to develop key elements of program expectations and users’ perceptions over time. Three themes transpired from the data: (1) Trustworthiness and timeliness of survivorship information and expert guidance, (2) Normalization of survivors’ experiences, and (3) Virtual program delivery issues. Some participants’ perceptions remained unchanged from pre-program expectations to post-program completion such as appreciating the efficiency of virtual delivery and “health safe” exchanges given the COVID-19 pandemic. In contrast, other perceptions became more polarized including drawbacks related to “more superficial” virtual connections and uneven topic relevance as the program evolved. Program participants appreciated timely information and support from volunteers and experts through virtual means and consecutive weekly sessions. Gauging participants’ perceptions across time also offer opportunities to adjust program content and delivery features. Future research should explore key program development strategies to ensure that cancer supportive programs are optimally person-centered, co-designed, and situation-responsive.

## 1. Introduction

As cancer-related mortality declines, cancer survivors are becoming an ever-growing group in need of multidimensional support and follow-up [1,2]. Survivorship typically refers to the post-treatment period [3], where affected individuals seek to adapt to a “new normal” while treatment-related issues wanes and transitions to everyday life activities ensue [4,5]. Survivorship usually involves new physical, emotional, or practical challenges [2,6,7]. Although these challenges can vary according to cancer diagnoses and stages, fatigue, uncertainty, and anxiety remain key concerns among survivors [5]. Survivors also often encounter difficulty seeking and receiving support for these concerns [8,9]. Consequently, their needs often go unmet [10]. Survivorship programs can facilitate these transitions, with community-based programs offering promise in terms of broader accessibility, affordability, and relevance [3,11,12,13].

Hope & Cope is a 400-plus volunteer community-based cancer organization affiliated with the Centre Intégré Universitaire de Santé et de Services Sociaux (CIUSSS) Centre Ouest (specifically the Jewish General Hospital) in Montreal, Quebec, Canada. Its primary mission is to assist individuals affected by cancer to regain a sense of control, hope, and enhanced well-being while decreasing social isolation [14]. With this aim in mind, Hope & Cope developed *Focus on the Future*, a six-week supportive program consisting of weekly two-hour sessions that provide knowledge and strategies to help cancer survivors adjust to life post-treatment. Each of the six sessions involves a trained volunteer moderator and expert guests (i.e., a dietician, a kinesiologist, a physiotherapist, a social worker, an occupational therapist, and an oncology nurse). The trained volunteer moderates and leads the first session on coping skills and relaxation and then co-leads the remaining five sessions according to the topics addressed. For instance, sessions two through six include presentations by healthcare professionals related to five core topics: (1) maintaining an active lifestyle, (2) managing energy, (3) nutrition, (4) establishing life goals beyond cancer treatment, and (5) anxiety and fear of cancer recurrence reduction. In response to the COVID-19 pandemic, the program was adapted from an in-person to a virtual format.

Cancer supportive programs are traditionally delivered in-person; however, there has been growing interest in providing support virtually because of its enhanced accessibility, contained cost, and convenience [15]. Since the early phases of the pandemic and associated physical distancing measures, supportive services and programs, including Hope & Cope, have rapidly adapted to provide virtual services [16]. In this public health emergency response context, it was essential that cancer survivors feel supported and continue to receive adequate care safely [17,18]. Furthermore, this format change required timely adaptation of service delivery given the intensified challenges experienced by individuals affected by cancer. Nonetheless, it is paramount that organizational stakeholders understand users’ perceptions of these new virtual services to inform optimal design, content, and delivery [19]. In addition, exploring users’ perceptions across pre-program enrolment, midway through, and post-program participation could further enhance the breadth and depth of this understanding [20].

The current study explored users’ evolving perceptions of “Focus on the Future” as a cancer survivorship program newly adapted to a virtual format. Studying users’ perceptions is necessary to ensure effective service delivery. Ordinarily, the acceptability of such programs should be determined before full implementation [20]; however, the transition to virtual delivery occurred quickly to ensure continuity of services at the onset of the pandemic. Consequently, cancer survivors’ perceptions of the program in its new virtual delivery format have yet to be explored. Therefore, the primary goal of this qualitative pilot study was to explore participants’ perceptions of the program across its delivery. Hence, the following questions guided this study:

1. What program expectations do participants hold before attending the virtual program?
2. What perceptions do participants have about the program at its mid-point?
3. How do participants view the program once it ends?

## 2. Materials and Methods

**Figure 1:**
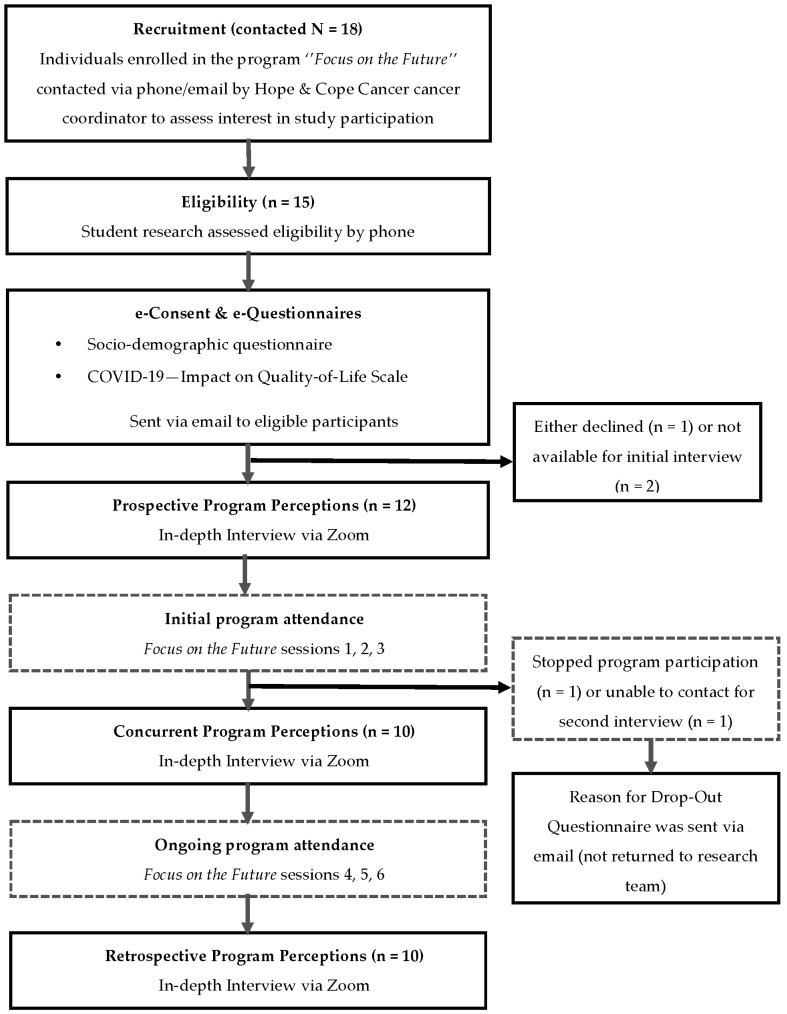
Data collection flowchart.

The present study applied an exploratory, descriptive qualitative design [21]. This design was chosen for its utility in providing descriptive accounts of understudied phenomena and informing the development of supportive programs [22,23]. A flow chart showing the study’s critical steps is presented in Figure 1. Using a purposive sampling approach between September and December 2021, individuals 18 years of age and over who had completed active cancer treatment within the last year and had registered for the program were recruited. Because the study’s sample was derived from a pre-existing program and fixed, practical considerations and judgment dictated a target sample of 10 to 12 participants [24]. Hope & Cope’s program coordinator contacted all 18 individuals registered for the fall 2021 English and French program versions and informed them of the study. The program coordinator told registered individuals that the study’s goal was to discuss survivors’ expectations and experiences in the upcoming virtual program to develop better programs tailored to their unique needs and preferences. Interested individuals were screened for eligibility and sent an e-consent form and a socio-demographic questionnaire via email. All questionnaires were completed online using Qualtrics, a secure electronic data capture system. Research assistants then contacted each participant to schedule individual interviews.

Study participants (n = 10) were interviewed three times: pre-program attendance (T1), mid-way through the program (T2), and after program completion (T3). These interview guides are available online as Supplementary Material Document S1. Interviews were conducted via Zoom’s video conferencing platform using audio only (i.e., cameras were turned off) and lasted between 45 and 60 min. The interview guide consisted of eight open-ended questions based on components of Sekhon and colleagues’ [20] theoretical framework for the acceptability of health-related interventions: (1) affective attitude, (2) burden, (3) ethicality, (4) intervention coherence, (5) opportunity costs, (6) perceived effectiveness, and (7) self-efficacy. At T1, example questions related to affective attitude, burden, and ethicality included “is there anything in particular that you expect to like about the program,” “how easy or difficult will it be to participate in the program,” and “to what extent do you think the program will be a good fit with your own values/core beliefs,” respectively. At T2, example questions for intervention coherence and opportunity costs included “to what degree do you understand how the program is benefitting you personally?” and “is there anything in particular that you are personally giving up or scarifying to participate in the program,” respectively. At T3, example questions linked to perceived effectiveness and self-efficacy include “to what extent do you think the program made a difference in the lives of people who used it” and “how confident are you that you were able to perform the tasks required by the program,” respectively. Study participants were assigned a numerical code by which their interview transcripts were identified to ensure anonymity. The interviews were transcribed verbatim and reviewed multiple times by the first author (A.R.). Next, A.R. openly coded the transcripts by highlighting specific sentences and words related to the phenomenon of interest, which were then grouped into themes using an inductive process. A.R. then created an initial coding structure with main themes and sub-themes. Subsequently, A.R. and C.G.L. validated the initial codes (approximately 20%) to ensure coding accuracy and reliability. Last, all authors discussed the coding structure and resolved interpretation discrepancies. Thematic analysis of the data revealed three overarching themes related to the perceived acceptability of the program [25].

## 3. Results

Participants’ socio-demographic characteristics are presented in Table 1. Participants were exclusively female, predominately Caucasian (i.e., 90%), and between 46 to 75 years old (M = 58.10, SD = 7.86). Findings were grouped into three themes that represent participants’ evolving program perceptions: (1) Trustworthiness and timeliness of survivorship information and expert guidance post-treatment completion, (2) normalization of survivors’ experiences, and (3) virtual program delivery issues. These three themes are described in detail herein.

### 3.1. Theme 1: Trustworthiness and Timeliness of Survivorship Information and Expert Guidance

All participants felt uncertain about the program’s purpose and how it might be beneficial. At T1, participants wanted to receive clear, trustworthy information to help them adapt to treatment completion. One participant hoped the program would allow her “to see how others are living with cancer, if there are things that you can learn from those experiences, and if I can support someone else [Translated from French].” (P7) Seven participants mentioned feeling abandoned and uncared for once their cancer treatment ended. They noted, at present, that they rarely met with their oncologists and struggled to receive answers to their questions from the cancer team. Moreover, they expressed uncertainty regarding whether their ongoing health issues (e.g., fatigue or loss of sensation) were normal and the best course of action for health management. Three participant statements include:

“Many people feel a little bereft, as though they’re sort of plunked into another universe, where they had all of this care and then there’s very little follow-up. And yet they still are suffering from the after-effects of chemotherapy.”

(P4)

“But in oncology, when it has been five minutes in the office, they [oncologists] quickly rush you out, they have other patients. Especially when it’s very busy [Translated from French].”

(P1)

“When our treatment ends, the most difficult thing is that suddenly we have no one taking care of us. I went from having 3 or 4 hospital appointments per week to 0, or once every six months. That is stressful [Translated from French].”

(P6)

Furthermore, five participants attempted to obtain information about how to best preserve health after cancer. Unfortunately, such efforts tended to generate more questions than answers, with ensuing skepticism concerning the validity of the information they found on their own. As one participant put it, “I bought myself 12 books [on cancer], and not one of them said the same things as the others. They contradicted each other [Translated from French].” (P1)

Participants thought the healthcare professionals provided reliable information when interviewed at T2 and T3. One participant noted, “We really appreciate the fact that each talk is given by an expert who knows what she’s talking about.” (P4) However, the first session, which a trained volunteer-led, was received with less enthusiasm. A participant stated: “I wished it had been more developed; it was a volunteer. I think it should be someone more professional because they are naming ways […] we can use to calm anxiety.” (P5) Nevertheless, participants welcomed the variety of topics and their relevance, with a participant stating, “it would be impossible for anyone to come to Focus on the Future and not take something away.” (P3)

Four participants were already familiar with the content covered; however, the depth of the information reinforced and validated their knowledge and encouraged them to start applying healthier habits. Conversely, one participant stated that she did not find the information helpful. As she explained:

“I was expecting to have information that I wouldn’t have had already, but I did my own research. So, the information that they gave was information that I had already looked up. I guess I was more prepared and that’s why maybe I found it boring.”

(P8)

The program also offered additional resources and services to participants. These included the possibility of booking individual consultations and an introduction to informational resources such as survivorship-related apps or websites. One participant stated, “We found out there were social workers, physiotherapists, psychologists, pain management specialists. Nobody had really told us about these different specialists [during treatment] that we could call on or make appointments with.” (P4)

Although all but one participant appreciated the information provided, some felt they would have benefited from receiving it earlier. Three participants wished the program had been available immediately after their treatment ended or even during treatment. However, participants who were only a few months post-treatment found the program’s timing appropriate. A participant observed:

“I had done another session with Hope & Cope, a newly diagnosed program. We also had various professionals come in to talk to us. I think at that time, it was so overwhelming just being newly diagnosed and starting treatment and stuff. And at this point, being done treatment and having access to these speakers talk to us about OT, social work, nutrition, and all those things now was amazing. Because all I want to focus on now is taking care of myself and I don’t have the distraction of being in treatment.”

(P10)

### 3.2. Theme 2: Normalization of Survivors’ Experiences

Before attending the program, all participants welcomed the opportunity to connect with other cancer survivors to mainly compare how they were managing post-treatment. At T2, five participants found it easier to connect with “people who are going through similar things.” (P3) At T3, many participants were pleased with their newly found sense of community built over the six weeks. As one participant stated, “it makes us feel less alone [Translated from French].” (P6) Alternatively, two participants felt they did not connect well with the group because of differences in cancer or family situation. Despite this difference, at T3, all participants were comforted by their impression that other survivors were undergoing similar post-treatment issues. Two related participant statements include:

“I feel relieved, thanks to the community. They make me feel less alone in this world, especially after the treatments.”

(P5)

“[The program] gave me comfort and awareness that I’m not alone and that what I’m feeling is normal.”

(P10)

In addition, by the end of the program, three participants felt grateful for their situation relative to others. As one participant acknowledged, “I feel that when I see the other participants, I realize that my situation is relatively privileged [Translated from French].” (P7)

### 3.3. Theme 3: Virtual Program Delivery Issues

At T1, all participants welcomed the convenience of the virtual delivery format. Four participants indicated they would not have joined the program if not for virtual delivery. All participants stated they anticipated the primary benefit of online delivery to be time saved (e.g., not needing to commute or find parking). At T2 and T3, this benefit was also highlighted. As a participant noted:

“Sometimes the thought of having to get up, get dressed, get out the door, get into a car, get on a bus makes it difficult, and it can stop you from wanting to do things. But when all you have to do is turn on your computer and put a smile on your face, that makes it so much easier.”

(P3)

This participant also reported that another advantage of virtual attendance was that participants felt comfortable and safe, which she believed fostered greater participation. In her words, “because you’re on a screen, and we’re passing person to person, when it’s your turn to speak people tend to speak; where in a [in-person] group setting, people may hold back a little bit more.” (P3) In addition, participants found virtual delivery convenient because it did not exacerbate post-treatment fatigue and mitigated the risk of COVID-19 exposure.

Despite these expected and tangible benefits, participants also discussed the potential shortcomings of virtual delivery. For example, at T1, some participants anticipated that it would be more challenging to connect with others virtually because “you relate to someone better if you’re actually in the same room.” (P9) At T2 and T3, some participants reversed some of their earlier opinions regarding virtual delivery. For example, despite initial concerns, four participants felt that they had made meaningful connections by initiating private chat conversations or getting participants’ contact information to further discuss relevant topics outside of the program. Two participants reported:

“I know in the past, I said there’s no one-on-one connection, but by the last session, I was able to privately chat with other participants and that was great. So, I don’t really have any cons [about virtual delivery of the program] anymore.”

(P10)

“I really didn’t find that [meeting virtually] stopped us from communicating with other people. I think that since we weren’t too big of a group, it helped us talk and exchange. I think we would have done the same in-person [Translated from French].”

(P2)

Still, other participants felt uncertain whether in-person attendance may have facilitated enhanced interpersonal connections. A participant noted:

“People tell us that we’re social creatures. I wonder: Is socializing [online] really as good? It works, yes. But I don’t know if long-term it’s as good for us as in-person contact […] I wonder, if in-person, we would have developed more ties with the other participants [Translated from French].”

(P7)

At T3, some participants discussed the potential disadvantages of virtual formats. For instance, four participants stated that they would have preferred to complete the program in-person because of their diminished “Zoom tolerance” and the challenge of staying focused for two hours in a virtual setting. Similarly, at T1, a participant welcomed the idea of meeting virtually because she could stay home with her children. However, by T3, she thought that continuous accessibility to her children led to significant interruptions: “when you’re home, those distractions are still there.” (P8) Overall, even participants who initially would have preferred an in-person program believed that virtual delivery was better than no structured support at all: “If I have to choose between having none [support], or having Zoom, I by far prefer having Zoom [Translated from French].” (P6)

## 4. Discussion

This qualitative study explored participants’ expectations and perceptions of Focus on the Future, a new virtual supportive program tailored to the needs of cancer survivors. Participants’ perceptions were documented before the program started, midway through the program, and shortly after the program ended. Findings underscore that cancer supportive programs must be timely and responsive to the needs and preferences of their users to optimally serve individuals transitioning to a new phase in their cancer experience. Likewise, participants also expressed the need to receive psychosocial and informational support as soon as possible during the initial days or weeks following treatment completion. These findings align with those of Raphael, Frey, and Gott [26] concerning experiences of distress among cancer survivors related to unmet informational needs, suggesting that survivors require additional support post-treatment. These findings also demonstrate the importance of Loiselle and Brown’s [27] person-centered approach to psychosocial oncology practice, which emphasizes observation and real-time responsiveness to individuals’ needs and preferences. In addition, they also provide added support for Jefford et al.’s [28] recent call for more sustainable, supportive models that address survivors’ unmet needs.

Many participants also felt frustrated that they no longer knew where to receive follow-up care as their access to regularly scheduled treatment appointments had concluded. Accordingly, participants welcomed and conveyed confidence in the information provided by healthcare professionals and appreciated the rare opportunity to ask questions during these presentations. This finding is consistent with Kent et al.’s [29] observation that cancer survivors often lack the information resources needed to help them navigate the physical and psychosocial challenges resulting from earlier cancer diagnosis and treatment. Moreover, most participants also reported feelings of isolation and concerns about the future, replicating previous findings by Williams and Jeanetta [30]. Furthermore, the convenience of virtual delivery represented a prime factor contributing to the program’s acceptability. Specifically, participants reported saving significant time and energy by attending the program virtually as they did not need to travel. This finding aligns with a systematic review by Dilworth and colleagues [31], whereby they identified travel as a perceived barrier to accessing psychosocial oncology support.

Participants also reported that attending the program virtually reduced their anxiety about coming into contact with the COVID-19 virus, which may have played a role in their acceptability of virtual support. Indeed, the results of a survey on cancer services during the pandemic conducted by the Quebec Cancer Coalition [18] found that 77.4% of individuals living with cancer (n = 402) had received or been offered a virtual health professional consultation, with 73% reporting that they were either satisfied or very satisfied with this new virtual approach to service delivery. Hence, virtual support may have been viewed as a pandemic-appropriate format without significant disadvantages as participants did not report barriers such as limited access to technology, infrastructure issues, or privacy concerns [32,33]. The pandemic and rapid adoption of virtual solutions may have contributed to participants’ acceptance of the program’s delivery means, leaving questions about program acceptability post-pandemic.

Despite accessibility and convenience, once the virtual program ended, some participants continued to assert that they would have preferred in-person delivery. They reported being fatigued by virtual meetings over Zoom, while some felt that sessions should have been shortened. This is consistent with Shoshan and Wehrt’s [34] findings that virtual meetings can be experienced as more exhausting than other formats. The authors suggest that perceiving a session as overly lengthy could represent “Zoom” fatigue instead of sessions being objectively too long. Notably, our participants reported feeling less bothered by session length when they were more interested in the topic. In addition, participants suggested that moderators should more actively manage off-topic discussions to mitigate concerns about lengthy discussions. Several participants also noted that in-person support would have fostered more social connections. This acknowledges that despite the acceptability of a virtual format, some individuals may continue to favor in-person formats [15,35]. However, other participants described exerting additional efforts to forge connections whereby they could interact outside the virtual space. Such differences may be attributable to individual needs and preferences [36].

This study has several limitations. First, participants’ views of the program were explored during the pandemic. Such unprecedented circumstances potentially affected the program’s acceptability, given the general imperative for virtual service delivery. Second, the characteristics of individuals who enrolled in the program are potentially different from those who did not, thereby limiting the generalizability of these programs. Third, the sample was homogeneous (i.e., all women and 90% Caucasian) and views may have been different with a more diverse sample of participants. Last, program acceptability based on the types of cancer diagnosis, treatment, and stages was not explored.

Researchers can identify the population’s needs on key points along with the transition into survivorship and disseminate their findings to develop more targeted support. Moreover, socializing among participants should be actively encouraged, with interpersonal bonding exercises built into the initial stages of the program. In virtual programs, promoting chat features or integrating a socializing activity into sessions could help facilitate this task. Furthermore, an additional session, facilitated by a psychologist, could be added at the beginning of the program dedicated to group formation and cohesion. In addition, hybrid support (i.e., virtual and in-person) could be developed and tested for efficacy. Such hybrid models could balance the convenience of virtual delivery with the enhanced interpersonal intimacy of in-person modalities.

## 5. Conclusions

As the number of cancer survivors continues to grow significantly, so does the onus on identifying and addressing survivors’ unmet needs using innovative, person-centered, co-designed, and situation-responsive approaches. Recognizing the rapid proliferation, convenience, and sustainability of digital health solutions also means that continuous quality improvement measures, users’ involvement in their development, and systematic evaluation must be prioritized. This study’s findings suggest that a comprehensive virtual program that relies on trained volunteers and healthcare professionals can help to address the needs of participants. Future research would deepen our understanding of virtual support’s long-term and post-pandemic impact. In addition, ongoing program evaluation, initiated even before the program occurs, is key to inform and adjust program content and delivery in real-time, whether virtual or in-person.

## Figures and Tables

**Table 1:** Participant Characteristics.

Characteristic	n	%	M	SD	Range
**Biological Sex**					
Female	10	100.00			
**Age (Years)**			58.10	7.86	46–75
40–49	1	10.00			
50–59	4	40.00			
60–69	4	40.00			
70–79	1	10.00			
**Civil Status**					
Married/Common law	6	60.00			
Separated/Divorced	3	30.00			
Single	1	10.00			
**Currently Living with Someone**					
Yes	7	70.00			
No	3	30.00			
**Dependents**					
With children	10	100			
Number of children			2.00	1.00	1–4
Children living with participant			1.33	0.52	1–2
**Ethnicity**					
Caucasian	9	90.00			
Southeast Asian	1	10.00			
**Birthplace**					
Canada	6	60.00			
Germany	1	10.00			
Laos	1	10.00			
USA	1	10.00			
USSR	1	10.00			
**Highest Education Level Completed**					
Undergraduate	4	40.00			
Graduate	3	30.00			
Technical/Vocational school/Pre-university	2	20.00			
High school	1	10.00			
**Work Status**					
Disability/Sick leave	4	40.00			
Full time (30+ h/week)	3	30.00			
Part time (< 30 h/week)	0	0			
**Progressive Return to Work over 12 Weeks**	1	10.00			
Retired	2	20.00			
**How Would you Describe Yourself**					
Current person with cancer	5	50.00			
Former person with cancer	5	50.00			
**Primary Language**					
English	4	40.00			
French	5	50.00			
Lao	1	10.00			

## Data Availability

Data from this study are available from the corresponding author upon request.

## Supplementary Materials

The following supporting information can be downloaded at: https://www.mdpi.com/article/10.3390/curroncol29110664/s1, Document S1: Interview Guides.
