# Supplementary material for: Cancer Survivors’ Evolving Perceptions of a New Supportive Virtual Program
Source: Curr Oncol. 2022 Nov 5;29(11):8431–41. doi: 10.3390/curroncol29110664 (PMC9689835; doi:10.3390/curroncol29110664)
Supplement: Supplementary file 1 [file curroncol-29-00664-s001.zip › curroncol-1960178-supplementary.pdf]

Article: “Cancer Survivors’ Evolving Perceptions of a New Supportive Virtual Program”

Supplementary Materials

Document S1. Interview guides

**First Semi-Structured Interview Guide (Prospective Phase)**

You are currently enrolled in Hope & Cope’s Focus on the Future virtual program. In this study, we would like to explore with you, your views on this virtual program. If you are ok with this, I’d like to start by asking you a few questions about what you expect from this program.

There are no right or wrong answers. We are interested in knowing more about what you may anticipate the program to be like.

1) Affective Attitude	Is there anything in particular that *you expect to really like* about this upcoming program?
2) Burden	In your opinion, how easy or difficult *will it be* to participate in (The Upcoming Program)?
3) Effectiveness	To what extent do you think (The Upcoming Program) *will make* a difference in the lives of people taking part in it?
4) Opportunity Costs	Is there anything that you personally *will be giving up* or sacrifice to participate in (The Upcoming Program)?
5) Intervention Coherence	How do you think (The Upcoming Program) *will benefit* you personally?
6) Self-Efficacy	How confident are you that you *will be able* to perform the tasks required by (The Upcoming Program)?
7) Ethicality	In your opinion, to what extent do you think (The Upcoming Program) *will be* a good fit with your own values/core beliefs?
8) Virtual Pros and Cons	What do you *expect might be* the pros and cons of participating in the upcoming program virtually (as opposed to face-to-face)?

- *Would you like to share any additional thoughts, views, or suggestions about the upcoming program?*
- *Do you have any questions for us?*
- *Thank you so much for your participation in this first part of the study. I’ll contact you for the second interview to hear about your actual experience mid-way through the program.*

Second Semi-Structured Interview Guide (Concurrent Phase)

You are currently enrolled in Hope & Cope's Focus on the Future virtual program. In this study, we would like to explore with you, your views on this virtual program. If you are ok with this, I'd like to start by asking you a few questions about your experience of this ongoing program.

There are no right or wrong answers. We are interested in knowing more about how you experience this ongoing program.

1) Affective Attitude	Is there anything in particular that *you really like* about (The Ongoing Program)?
2) Burden	In your opinion, how easy or difficult *has it been* to participate in (The Ongoing Program)?
3) Effectiveness	To what extent do you think (The Ongoing Program) *is making* a difference in the lives of people taking part in it?
4) Opportunity Costs	Is there anything that you personally *are giving up* or scarifying to participate in (The Ongoing Program)?
5) Intervention Coherence	How do you think (The Ongoing Program) *is* benefiting you personally?
6) Self-Efficacy	How confident are you that you *are able* to perform the tasks required by (The Ongoing Program)?
7) Ethicality	In your opinion, to what extent do you think (The Ongoing Program) *is* a good fit with your own values/core beliefs?
8) Virtual Pros and Cons	What *are* the pros and cons of participating in the ongoing program virtually (as opposed to face-to-face)?

- *Would you like to share any additional thoughts, views, or suggestions about the ongoing program?*
- *Do you have any questions for us?*
- *Thank you so much for taking part in this second part of the study. I'll contact you for the final interview to hear about your overall experience after completing the program*

**Third Semi-Structured Interview Guide (Retrospective Phase)**

You recent completed Hope & Cope's Focus on the Future virtual program. In this study, we would like to explore with you, your views on this virtual program. If you are ok with this, I'd like to start by asking you a few questions about what your overall experience of the program.

There are no right or wrong answers. We are interested in knowing more about your overall experience of the program.

1) Affective Attitude	Is there anything in particular that *you really liked* about (The Recently Completed Program)?
2) Burden	In your opinion, how easy or difficult *was it* to participate in (The Recently Completed Program)?
3) Effectiveness	To what extent do you think (The Recently Completed Program) *made* a difference in the lives of people who took part in it?
4) Opportunity Costs	Is there anything that you personally had to *give up* or sacrificed to participate in (The Recently Completed Program)?
5) Intervention Coherence	How do you think (The Recently Completed Program) benefitted you personally?
6) Self-Efficacy	How confident are you that you *were able* to perform the tasks required by (The Recently Completed Program)?
7) Ethicality	In your opinion, to what extent do you think (The Recently Completed Program) *was* a good fit with you own values/core beliefs?
8) Virtual Pros and Cons	What *were* the pros and cons of participating in the recently completed program virtually (as opposed to face-to-face)?

- *Would you like to share any additional thoughts, views, or suggestions about the recently completed program?*
- *Do you have any questions for us?*
- *Thank you so much for taking part in this final part of the study.*
